# mTOR Modulates the Endoplasmic Reticulum Stress-Induced CD4^+^ T Cell Apoptosis Mediated by ROS in Septic Immunosuppression

**DOI:** 10.1155/2022/6077570

**Published:** 2022-07-23

**Authors:** Hao Wang, Jianwei Chen, Guangxu Bai, Wen Han, Ran Guo, Na Cui

**Affiliations:** ^1^Department of Critical Care Medicine, Beijing Jishuitan Hospital, Beijing 100035, China; ^2^Department of Critical Care Medicine, State Key Laboratory of Complex Severe and Rare Diseases, Peking Union Medical College Hospital, Chinese Academy of Medical Science and Peking Union Medical College, Beijing 100730, China

## Abstract

**Introduction:**

When sepsis attacks the body, the excessive reactive oxygen species (ROS) production can result to endoplasmic reticulum stress (ERS) and eventually cause lymphocyte apoptosis. The mammalian target of rapamycin (mTOR) is essential for regulating lymphocyte apoptosis; we hypothesized that it mediates CD4^+^ T cell apoptosis during ROS-related ERS.

**Method:**

We, respectively, used ROS and ERS blockers to intervene septic mice and then detected ERS protein expression levels to verify the relationship between them. Additionally, we constructed T cell-specific mTOR and TSC1 gene knockout mice to determine the role of mTOR in ROS-mediated, ERS-induced CD4^+^ T cell apoptosis.

**Results:**

Blocking ROS significantly suppressed the CD4^+^ T cell apoptosis associated with the reduction in ERS, as revealed by lower levels of GRP78 and CHOP. ERS rapidly induced mTOR activation, leading to the induction of CD4^+^ T cell apoptosis. However, mTOR knockout mice displayed reduced expression of apoptotic proteins and less ER vesiculation and expansion than what was observed in the wild-type sepsis controls.

**Conclusion:**

By working to alleviate ROS-mediated, ERS-induced CD4^+^ T cell apoptosis, the mTOR pathway is vital for CD4^+^ T cell survival in sepsis mouse model.

## 1. Introduction

An epidemiologic survey in 2020 reported that although the average worldwide death rate from sepsis is around 15%–30%, it is even higher in some underdeveloped regions [1]. Sepsis causes countless deaths and imposes huge economic burdens on countries [2]. Over the years, research has determined that immunosuppression is one of the major reasons for high mortality in patients with sepsis [3^,^^4^]. Abnormal immune cell death is an important cause of immunosuppression in sepsis, and excessive apoptosis of lymphocytes is one such type of immune cell death [5^,^^6^].

In recent years, researchers have discovered a new type of apoptosis caused by endoplasmic reticulum stress (ERS), and this type of apoptosis has been named the third apoptotic pathway. In pathological conditions such as infection, sepsis, and hypoxia, cell energy metabolism disorders induce reactive oxygen species (ROS) production, which renders the endoplasmic reticulum (ER) unable to correctly synthesize proteins [7]. Large amounts of unfolded or misfolded proteins accumulate, and this leads to ERS. When ERS persists and cell homeostasis cannot be recovered, cell apoptosis will occur [8]. Therefore, preventing ERS may relieve the inflammatory state and damage to tissues and organs [9, 10].

As essential immune system components, reduced numbers of CD4^+^ T lymphocytes will further aggravate immune deficiency in the body. Reduced CD4^+^ T cell absolute number is an independent risk factor that influences the outcome of sepsis patients [11]. Mammalian target of rapamycin (mTOR) is a serine/threonine protein kinase, which played significant role in regulating various cellular metabolic activities including cell energy metabolism and protein synthesis. One important role that it plays is in regulating cell autophagy and apoptosis. One important role that it plays is in regulating cell autophagy and apoptosis. In addition, mTOR complex 1 (mTORC1) responds to low ATP levels or a hypoxic unstable intracellular environment. Tuberous sclerosis complex 1 (TSC1) functions as a GTPase-activating protein to activate mTORC1 [12]. We have previously confirmed that mTOR is involved in the immunosuppression of sepsis and that the apoptosis of CD4^+^ T cells can be attenuated by mTOR intervention [13, 14]. However, the region regulating CD4^+^ T cell apoptosis upstream of mTOR has yet to be studied, and this knowledge gap in the mTOR pathway awaits elucidation. Interestingly, emerging literature has indicated that the mTOR signaling acts downstream of the ER and mediates ERS-induced apoptosis of pulmonary vascular endothelial cells [15]. We speculate that mTOR might also function as import regulator during the process of CD4^+^ T cell apoptosis caused by ERS in sepsis.

We hypothesized that mTOR is involved in the regulation of CD4^+^ T cell apoptosis triggered by ROS-related ERS. The cecal ligation and puncture (CLP) model is referred to be the “gold standard” for the induction of polymicrobial sepsis in experimental settings to research the fundamental mechanisms of sepsis. Besides, the cytokine profile of the CLP model is similar to that seen in human sepsis where there is increased lymphocyte apoptosis [16]. To test this hypothesis, we constructed an mTOR knockout mouse sepsis model to study the role of mTOR. We used this model to investigate whether mTOR could be pharmacologically used to regulate CD4^+^ T cell apoptosis thereby enhancing the immunity and improving the prognosis of patients with sepsis.

## 2. Materials and Methods

### 2.1. Mice

Male C57BL/6N mice were housed in a pathogen-free animal house with comfortable environment (room temperature 25 ± 1°C, 12 h day/night cycle). We used 16-18 g healthy male C57BL/6N mice (aged 4-5 weeks) to establish the sepsis model. Lck-cre mice were generated by crossing TSC1loxp/loxp and mTORloxp/loxp mice with mice expressing Cre recombinase. Lck-cre mTORloxp/loxp (lck-mTOR) and Lck-cre TSC1loxp/loxp (lck TSC1) mice obtained, Lck-cre-negative mTOR loxp/loxp mice were their corresponding controls. Lck-mTOR mice were used in the Lck-mTOR+CLP group (*n* = 6). The Lck-TSC1 mice were used in the Lck-TSC1+CLP group (*n* = 6). 30 lck-mTOR mice (*n* = 6 per group) were allocated to each group: WT, CLP, CLP+NAC, CLP+CIRP-Ab, and CLP+4-PBA group.

### 2.2. Sepsis Model

We establish a midgrade sepsis model according to the protocol as previously described [16].

### 2.3. Drug Administration

In treatment groups, CIRP-Ab (C23. 8 mg/kg BW. GenScript: Nanjing, China) was administered by intravenous tail injection after 2 h of the CLP operation. N-Acetyl-L-cysteine (NAC, 150 mg/kg BW, Beyotime: Shanghai, China) and 4-phenylbutyric acid (4-PBA. 40 mg/kg BW, TargetMol, USA) were administered intraperitoneally 60 min before surgical procedure. Besides, DMSO (10% DMSO, 4 ml/kg BW) was administered intraperitoneally to mice in the control group. After 18 h of the treatment, all mice were killed, and spleens were collected immediately. The experiments were carried out under strict guidance and principles according to the guidelines of PUMCH Clinical Laboratory. JS-1170 was the approval number for the study from the Ethics Committee at Peking Union Medical College Hospital (Beijing, China).

### 2.4. Spleen Tissue Single-Cell Suspension

The harvested spleens were minced, and the homogenate was then passed through a 40 *μ*m cell strainer. The obtained cells were lysed in red blood cell lysis buffer, followed by being transferred to 15 ml centrifuge tubes. To make single-cell suspension, samples were centrifuged at 1500 rpm for 10 min and resuspended in 5 ml of PBS.

### 2.5. Lymphocyte Purification

Mouse spleen lymphocytes were purified using the lymphocyte separation kit (Solarbio, Beijing, China.). Briefly, 5 ml of lymphocyte separation medium was gently added to 5 ml spleen tissue single-cell suspension; after centrifugation at 800*g* for 20 minutes, lymphocytes were transferred into a new 15 ml centrifuge tube. Following washing with 10 ml washing solution and centrifugation at 250*g* for 10 min, the separated spleen lymphocytes were suspended with PBS.

### 2.6. Sorting CD4^+^ T Cells

CD4^+^ T cells were isolated from lymphocytes by negative magnetic bead sorting using magnetic microbeads (Miltenyi Biotec, Bergisch Gladbach, Germany). Briefly, we stained splenocytes with biotin-conjugated anti-CD4 antibody for 30 minutes and then washed with PBS for 5 min 3 times; we next incubated the cells for 15 minutes with magnetic streptavidin. Afterward, we used the CD4^+^ T cells for apoptosis array, ROS detection, RT-PCR, western blotting, and transmission electron microscopy.

### 2.7. Western Blot

CD4^+^ T cells lysate was prepared by lysing in RIPA buffer containing protease inhibitors. Protein concentrations were estimated (BCA protein assay kit). After that, the protein samples were loaded onto each lane by SDS-PAGE for electrophoresis and transferred to 0.45 *μ*M PVDF membrane (Millipore, MA, USA). Next, PVDF membranes were blocked with 5% skimmed milk in TBS-Tween for 60 min, followed by incubation with antibody at 4°C for 12-16 h. The following primary antibodies are as follows: anti-mTOR (Cat#AF6308, dilution rate 1 : 1000), anti-P-mTOR (Cat#AF3308, 1 : 1000), anti-P-p70s6k (Cat#AF3228, 1 : 1000), anti-p70s6k (Cat#AF6226, 1 : 1000), anti-4EBP (Cat#AF6432, 1 : 1000), anti-caspase-3 (Cat#AF6311, 1 : 1000), anti-Bcl-2 (Cat#AF6139, 1 : 1000), anti-Bax (Cat#AF0120, 1 : 1000), anti-GRP78 (Cat#AF5366, 1 : 1000), anti-CIRP (Cat#DF2643, 1 : 1000), anti-CHOP (Cat#DF6025, 1 : 1000), anti-CIRP (Cat#AF5366, 1 : 1000), and anti-actin-*β* (Cat#AF7018, 1 : 3000) were purchased from Affinity Biosciences (Jiangsu, China). Anti-P-4EBP (anti-eIF4EBP1, ab27792) was from Abcam (CA, USA). After washing with TBS-Tween 3 times, the membranes were incubated with a goat anti-rabbit IgG antibody (1 : 5000, Affinity Biosciences) at 25°C for 1 h, and the chemiluminescence signals were detected using an electrochemiluminescence detection system. Band densities were quantified by the *ImageJ software*.

### 2.8. Apoptosis Array

Cell apoptosis array was carried out following the protocol of the apoptosis detection kit (BD Pharmingen, USA). Cells were resuspended at 1 × 10 cells/ml in binding buffer and incubated with Annexin V-FITC and PI for 15 minutes in the dark before analyzing cells on a flow cytometer. FCS files were analyzed using the FlowJo software.

### 2.9. Intracellular ROS Detection

The level of ROS in CD4^+^ T cells was evaluated according to the protocol of ROS detection kit (Biyuntian, Shanghai, China). DCFH-DA and CD4^+^ T cells were mixed and incubated at 37°C for 20 minutes. ROS levels were then measured by flow cytometry.

### 2.10. RNA Extract and RT-PCR

CD4^+^ T cells were collected and RNA were harvested in TRIzol reagent (Tiangen, Beijing, China). RNA was then reverse transcribed using a PrimeScript RT reagent kit. qPCR was performed with SYBR Premix EX Taq™ II in ABI 7500 real-time PCR system (Applied Biosystems, USA). Primers are as follows: Bim sense: 5′-GAGATACGGATTGCACAGGA-3′, Bim antisense: 5′ -TCAGCCTCGCGGTAATCATT-3′; *β*-actin sense: 5′-ACTGGGACGACATGGAGAAG-3′, *β*-actin antisense: 5′-GGGGTGTTGAAGGTCTCAAA-3′. Data analysis used the 2-*ΔΔ*Ct method and normalized to the housekeeping *β*-actin gene.

### 2.11. Conventional Reverse Transcriptase Polymerase Chain Reaction

To detect mTOR and TSC1 expression levels, cDNA was amplified through a 32-cycle PCR: 95°C for 30 sec, followed by 32 cycles of 55°C for 30 sec and 30 sec at 72°C. Agarose gel electrophoresis method (1.5% agarose gel) was conducted to evaluated PCR products. The size of the PCR products was checked by 2 kb DNA Ladder (3427Q, Takara, Japan).

### 2.12. Transmission Electron Microscopy

CD4^+^ T cells were fixed with 2.5% glutaraldehyde and stored at 4°C. Next, the samples were fixed with osmic acid at 4°C for 3 h after washing with phosphate buffer. They were then dehydrated stepwise in increasing concentrations of ethanol, infiltrated in Spurr resin overnight, embedded in Spurr resin, and cured in a 70°C oven for 24 h. Finally, thin sections (90 nm) were made and stained with uranyl acetate and lead citrate; the sections were afterward viewed with a transmission electron microscope. Representative images from randomly selected fields under the microscope are shown.

### 2.13. Statistical Analysis

The number of mice used for the experiments comprised at least six mice per treatment. All data in this study were derived from 3 or more independent experiments. The data are presented as the means ± SD. The results were analyzed using the GraphPad Prism 8.0 software. One-way ANOVA with Tukey's post hoc or Dunnett's post hoc analysis was done to test multiple comparisons. Kaplan-Meier with log rank test was used for survival analysis. In the case of western blot, apoptosis, and electron microscope, one representative set of data is shown. *P* values < 0.05 were considered statistically significant. ^∗^*P* ≤ 0.05, ^∗∗^*P* ≤ 0.01, ^∗∗∗^*P* ≤ 0.001, and ^∗∗∗∗^*P* ≤ 0.0001.

## 3. Results

### 3.1. Genotype Identification of Conditional Gene Knockout Mouse Used in CLP Model and Survival Observation

**Figure 1:**
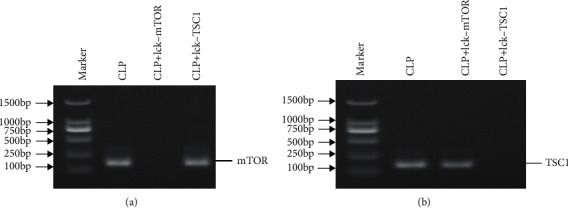
mTOR and TSC1 mRNA expression in CD4^+^ T cells in CLP, lck-mTOR, and lck-TSC1 mice. Agarose gel electrophoresis of colony PCR (a) mTOR and (b) TSC1 genotyping.

**Figure 2:**
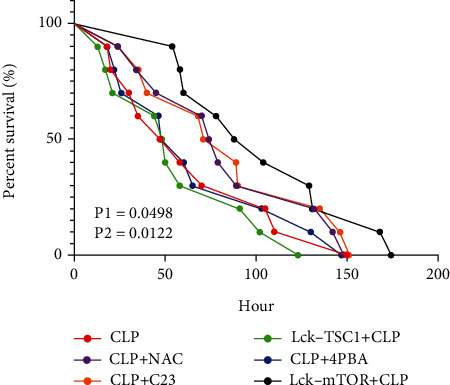
Survival curves. Survival rates between CLP, CLP+NAC, CLP+C23, CLP+4-PBA, Lck-TSC1+CLP, and Lck-mTOR+CLP groups. During the first 18 hours after CLP operation, the survival conditions were regularly monitored every 2 hours and then observed every 6 hours. Median survival time of CLP was 64 h, and LCK-TSC1+CLP group was 56 h, while Lck-mTOR+CLP had a significantly prolonged median survival time of 104 h (Kaplan-Meier with log rank test). *n* = 10-14 mice per group. *x*-axis: survival time; *y*-axis: survival rate. P1 = LCK-TSC1+CLP vs. Lck-mTOR+CLP; P2 = CLP vs. Lck-mTOR+CLP. NAC = N-acetyl-L-cysteine; 4-PBA = sodium phenylbutyrate; C23 = CIRP-Ab; CIRP = cold-inducible RNA-binding protein.

To determine the role of mTOR in ERS-induced CD4^+^ T cell apoptosis, we constructed a sepsis model with T cell-specific mTOR and TSC1 gene knockout mice. The genotypes of the experimental mice were confirmed by RT-PCR (Figure 1). The expression of mTOR mRNA was significantly decreased in lck-mTOR CD4^+^ T cells, while the expression of TSC1 mRNA was significantly decreased in lck-TSC1 CD4^+^ T cells. RT-PCR results confirmed the establishment of T cell-specific mTOR/TSCI-KO gene knockout mouse. Survival rates between groups were also analyzed. Compared to the CLP group, the LCK-TSC1+CLP group showed higher mortality, whereas the Lck-mTOR+CLP group had significantly lower mortality (Figure 2).

### 3.2. Endoplasmic Reticulum Stress-Induced Apoptosis Leads to CD4^+^ T Cell Depletion in Sepsis

**Figure 3:**
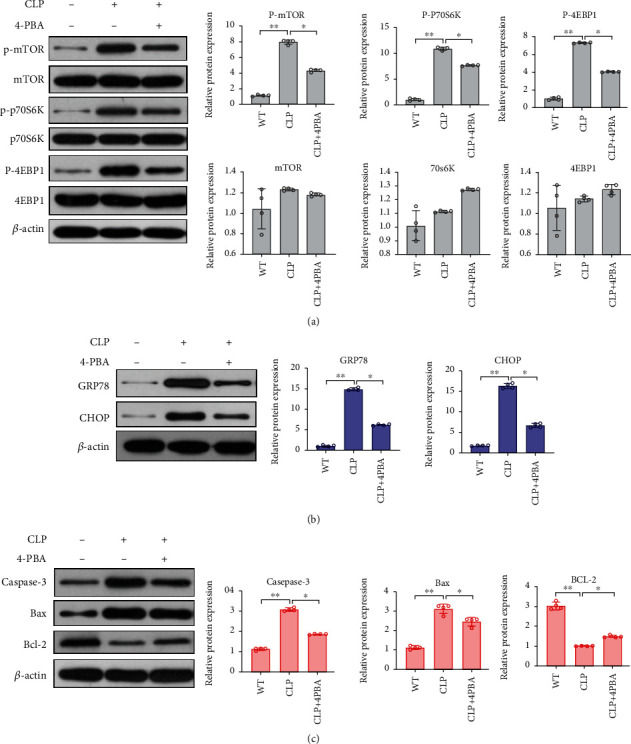
Expression levels of mTOR pathway proteins, ERS-associated proteins, and apoptosis-associated proteins in WT, CLP, and CLP+4-PBA mouse groups. After purifying the CD4^+^ T cells from mouse spleen lymphocytes, whole cell lysates were assessed for the protein expression of (a) patterns of mTOR pathway proteins, including mTOR, P-mTOR, downstream effectors p70s6k, p-p70s6k, 4EBP, and P-4EBP; (b) ERS-associated proteins, including GRP78 and CHOP; (c) apoptosis-associated proteins, including caspase-3, Bax, and Bcl-2. The protein expression was detected by immunoblotting. Data are mean ± SD. *n* = 4 biologically independent experiments (one-way ANOVA Tukey's post hoc test). ^∗^*P* < 0.05, ^∗∗^*P* < 0.01, and ^∗∗∗^*P* < 0.001.

**Figure 4:**
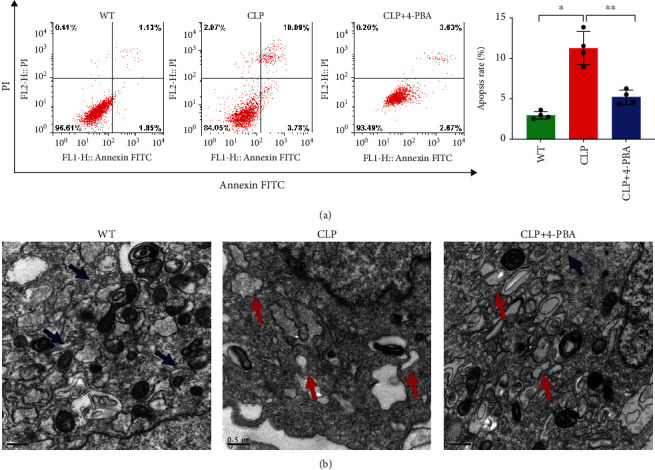
ERS-induced apoptosis leads to CD4^+^ T cell apoptosis in CLP mice. Apoptosis in CD4^+^ T cells from WT, CLP, and CLP+4-PBA mice was assessed by flow cytometry analysis. Apoptosis ratios are the early apoptosis percentage plus the late apoptosis percentage (a). *x*-axis: V-FITC; *y*-axis: PI. Left panel: gating strategy for apoptosis cells; right panel: percentage of apoptotic cells in the left panel. Graphs show means ± SD, four to six mice per group. Representative images of ER in CD4^+^ T cells from WT, CLP, and CLP+4-PBA mice, as observed by electron microscopy (b). Data are mean ± SD, *n* = 4 (one-way ANOVA Dunnett's post hoc test). Blue arrows represent normal-sized ER. Red arrows represent dilation and vesiculation of the ER. Scale bars = 0.5 *μ*m. FITC: fluorescein isothiocyanate; PI: propidium iodide.

**Figure 5:**
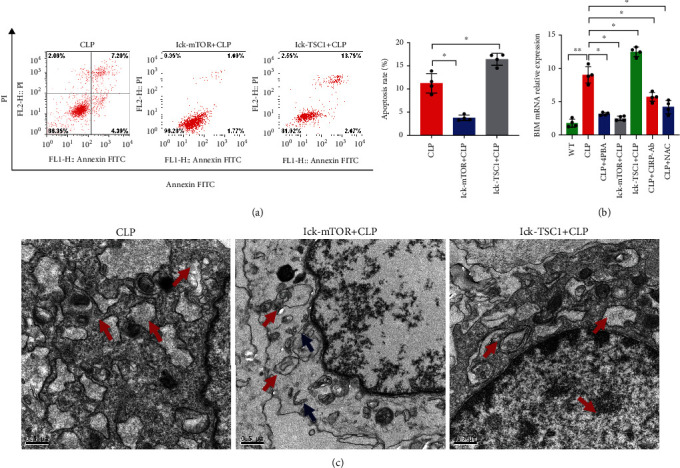
Role played by mTOR in ERS and CD4^+^ T cell apoptosis. CD4^+^ T cell apoptosis percentage was assessed by flow cytometry (a). Left panel: gating strategy for apoptosis cells; right panel: percentage of apoptotic cells in the left panel. Graphs show means ± SD, four mice per group. mRNA expression level of BIM in CD4^+^ T cells was analyzed by RT-PCR (b). Representative images of ER in CD4^+^ T cells from CLP, LCK-TSC1+CLP, and Lck-mTOR+CLP mice, as observed by electron microscopy (c). Data are mean ± SD, *n* = 4, analyzed using one-way ANOVA. Blue arrows represent the normal-sized ER. Red arrows represent dilation of the ER. Scale bars = 0.5 *μ*m.

By constructing a mouse sepsis model, we next investigated the relationship between sepsis, ERS, and CD4^+^ T apoptosis. In comparison to the WT group, the CLP group exhibited statistically higher GRP78 and CHOP expression levels (Figure 3(b)). Electron microscopy also showed that significant ERS manifestations, such as dilatation and vesiculation of the ER structures, had occurred in the CLP group (Figure 4(b)). Furthermore, western blots showed that Bax, caspase-3, and BIM expression levels were upregulated in the CLP group, while BCL-2 was downregulated (Figure 3(c) and Figure 5(b)). As showed in the flow cytometry results, compared with other groups, the CLP group has higher apoptosis rate of CD4^+^ T cells (Figure 4(b)).

The relationship between ERS and CD4^+^ T cell apoptosis was studied using 4-PBA since it blocks ERS. Compared with the CLP group, GRP78 and CHOP expression levels were reduced in the CLP+4-PBA group (Figure 3(b)), and less serious ERS was observed by electron microscopy (Figure 4(b)). Compared with other groups, the CLP+4-PBA group displayed decreased p-mTOR, p-p70s6k, p-4EBP1, and CHOP expression and apoptosis-related proteins such as BIM, Bax/Bak, and caspase-3 and an upregulated Bcl-2 expression (Figures 3(a)–3(c)). The flow cytometry results showed that in the CLP+4-PBA group, fewer apoptotic CD4^+^ T cells were found than in the other groups (Figure 4(a)). These results indicate that sepsis induces ERS and then causes CD4^+^ T cell apoptosis, and that ERS activates mTOR and blocking ERS can alleviate CD4^+^ T cell apoptosis.

### 3.3. mTOR Is Involved in ERS-Induced CD4^+^ T Cell Apoptosis

**Figure 6:**
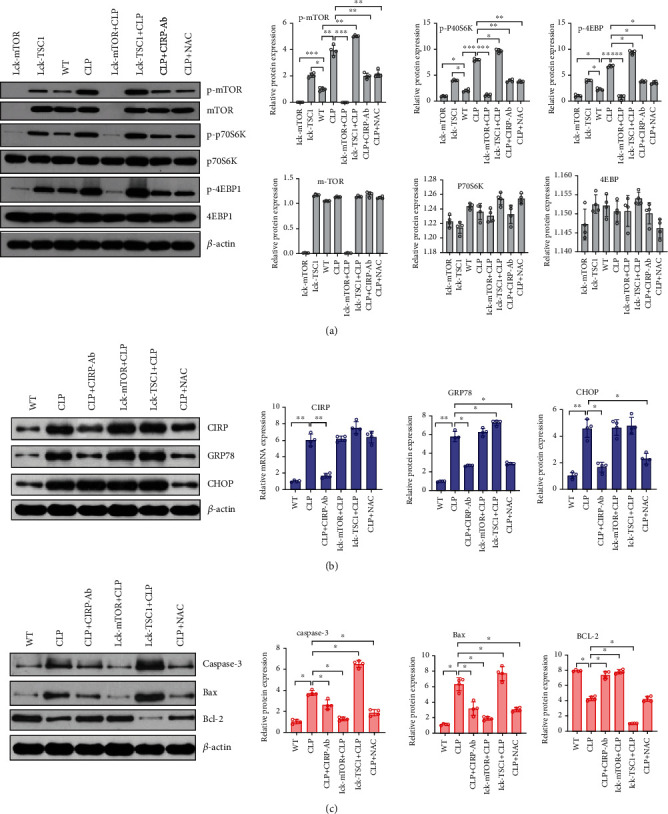
CD4^+^ T cells were assessed for the expression of mTOR pathway-associated proteins, ERS-associated proteins, and apoptosis-associated proteins in WT, LCK-mTOR, LCK-TSC1, CLP, CLP+CIRP-Ab, CLP+NAC, LCK-TSC1+CLP, and Lck-mTOR+CLP mouse groups. Data are mean ± SD. Number of mice per group = 4 (one-way ANOVA Tukey's post hoc test). CD4^+^ T cells were purified from CIRP-Ab-treated mice, NAC-treated mice, TSC1 knockout mice, and mTOR mouse spleen lymphocytes. Total proteins were western blotted to identify the expression patterns of mTOR pathway proteins (P-mTOR, mTOR, p70s6k, p-p70s6k, P-4EBP, and 4EBP) (a); CIRP and ERS-associated proteins (GRP78 and CHOP) (b); and apoptosis-related proteins (caspase-3, Bax, and Bcl-2) (c).

The p-mTOR expression levels were found to have decreased significantly in the CD4^+^ T cells from the lck-mTOR mice and increased in the lck-TSC1 mice. In contrast, in the lck-mTOR+CLP and lck-TSC1+CLP groups, GRP78 and CHOP levels did not differ significantly (Figures 6(a) and 6(b)). We next examined what role mTOR plays in CD4^+^ T cell apoptosis. We found that the expression levels of Bax, caspase-3, and BIM were downregulated, and the expression level of Bcl-2 increased.

In our study, we found that Bax, caspase-3, and BIM expression levels decreased, whereas Bcl-2 expression increased, and the CD4^+^ T cell apoptosis rate decreased significantly in the lck-mTOR sepsis mice. However, Bax, caspase-3, and BIM were upregulated, the expression levels of Bax, caspase-3, and BIM were upregulated, the BCL-2 expression level was downregulated, and the CD4^+^ T cell apoptosis rate significantly increased in the lck-TSC1 sepsis mice (Figure 5(b) and Figure 6(c)). The above results suggest that ERS activates mTOR, thereby causing apoptosis, and that blocking mTOR can alleviate ERS-related CD4^+^ T apoptosis.

### 3.4. The Role Played by ROS in ERS, mTOR Expression Regulation, and CD4^+^ T Cell Apoptosis

**Figure 7:**
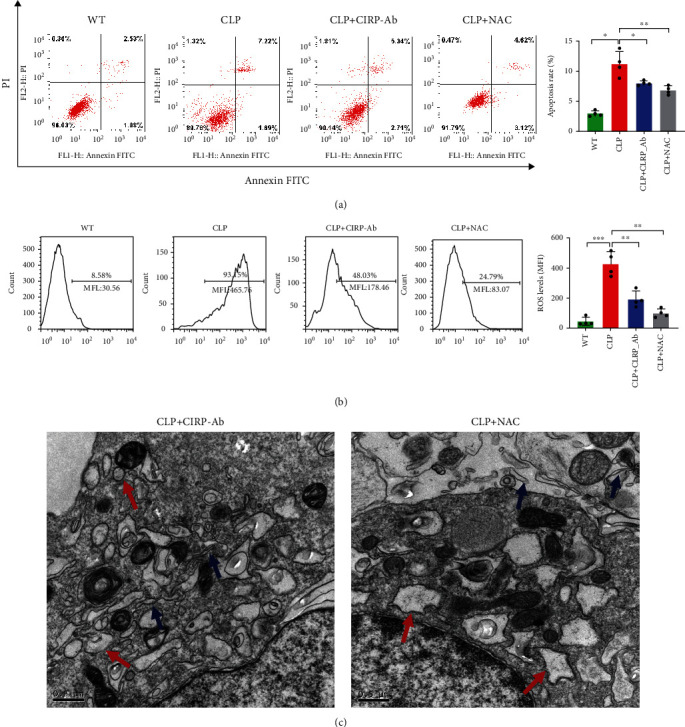
Role played by ROS in ERS and CD4^+^ T cell apoptosis. The apoptosis percentage of CD4^+^ T cell from WT, CLP, CLP+CIRP-Ab, and CLP+NAC mice was flow cytometrically determined (a). Left panel: gating strategy for apoptosis cells; right panel: percentage of apoptotic cells in the left panel. Data represent the mean ± SD. Graphs show means ± SD, four mice per group. CD4^+^ T cells were treated with CM-H2DCFDA for 45 min prior to ROS analysis by flow cytometry (b). Representative images of ER in CD4^+^ T cells from CLP, CLP+CIRP-Ab, and CLP+NAC mice, as observed by electron microscopy (c). Data are mean ± SD of 4 independent experiments (one-way ANOVA Tukey's post hoc test). Blue arrows indicate normal-sized ER. Red arrows indicate dilatation and vesiculation of the ER. Magnification, ×20,000; scale bars = 0.5 *μ*m.

We focus on the effects of ROS on ERS and CD4^+^ T cell apoptosis in the mouse sepsis model by using NAC to block ROS. Mice were first injected intraperitoneally with NAC 1 h before surgery. Thereafter, the effects of NAC on ERS and apoptosis in splenic CD4^+^ T cells from the mice were examined postsurgery. In comparison with the CLP group, the CLP+CIRP-Ab group had a lower ROS level (Figure 7(b)) and lower CHOP, GRP78, P-mTOR, P-4EBP, and p-p70s6k expression levels (Figures 6(a) and 6(b)). Furthermore, in the CLP+CIRP-Ab group, BIM expression was significantly downregulated, Bcl-2 was upregulated, and Bax and caspase-3 expression levels were downregulated (Figure 5(b) and Figure 6(c)). CHOP and GRP78 expression was decreased, and they also significantly differed from some of the other groups (Figure 6(b)). Furthermore, electron microscopy results showed fewer signs of ERS within the CD4^+^ T cells from the CIRP-Ab+CLP mouse group compared with those from the CLP group (Figure 7(c)); a lower percentage of apoptotic CD4^+^ T cells was also recorded in the former group (Figure 7(b)).

CIRP, a damage-associated molecular pattern (DAMP) molecule, is an important upstream molecule of ROS [17]. To further explore the role played by ROS, we blocked CIRP expression in mice using a CIRP-Ab and examined its effects on ROS level, ERS, and apoptosis. The ROS level of the CLP+CIRP-Ab group was lower than that of the CLP group (Figure 7(b)) and lower CHOP, GRP78, P-mTOR, P-4EBP, and p-p70s6k expression levels (Figures 6(a) and 6(b)). Furthermore, in the CLP+CIRP-Ab group, BIM expression was decreased, Bcl-2 was increased, Bax and caspase-3 were downregulated (Figure 4(b) and Figure 6(c)), and CHOP and GRP78 expression was downregulated and significantly different (Figure 6(b)). Furthermore, the electron microscopy results showed fewer signs of ERS within the CD4^+^ T cells from the CIRP-Ab+CLP mouse group compared with those from the CLP group (Figure 7(c)); a lower level of apoptotic CD4^+^ T cells was also recorded in the former group (Figure 7(b)).

## 4. Discussion

Sepsis is a type of life-threatening organ dysfunction feature based on early-onset inflammatory storm and late-onset immunosuppression [18]. Despite the advancement of many medical technologies such as early 6-hour sepsis bundle therapy, immunotherapy, and advanced organ function support, the prognosis for sepsis patients is far from ideal. One reason for this is unrelieved immunosuppression in those with deteriorating infections [5]. Studies by Inoue et al. found that excessive T lymphocyte apoptosis is closely associated with immunosuppression and is apparently linked with the prognosis for sepsis [19^,^^20^]. The present study, together with our previous research on the abnormal apoptosis mechanism in CD4^+^ T cells, revealed the complete process underlying sepsis-related CIRP–ROS-ERS–mTOR–CD4^+^ T cell apoptosis. This is the first time that mTOR proved to be involved in the CD4^+^ T cell apoptosis caused by ROS-mediated ERS in sepsis. We found that inhibiting mTOR reduced the high apoptotic rate of CD4^+^ T cells in septic host and ultimately improved the prognosis for these mice.

The ER is a vital organelle involved in protein synthesis and secretion. It is also responsible for protein translation, processing, and modification. When sepsis, infection, and other diseases attacked the body, there is a mass of unfolded or misfolded proteins accumulated in cells. This accumulation disrupts homeostasis in the ER and causes ERS [21]. Moreover, there are many diseases associated with ERS, including sepsis, cancer, and diabetes [22–24]. The use of drugs or gene therapy strategies to inhibit ERS has successfully improved the pathological characteristics of such stress, thereby imparting therapeutic effects on the target organs [10, 24–26]. Therefore, lymphocyte ERS is expected to serve as a new therapeutic target to improve immune function in the sepsis patient. ROS accumulation is a sign of cell energy metabolism disorders. Under cell stress, hypoxia, and other oxidative stimuli, intracellular ROS production greatly increases. Excessive ROS can cause cell damage, cell metabolic disorders, even cell apoptosis [27]. Many recent studies have shown that when the cell energy transfer process is impeded, and the accumulation of excessive ROS will also seriously affect the cell's protein synthesis function; this situation inevitably causes ERS, further leading to cell death [28^,^^29^]. Therefore, ROS and ERS are causal with respect to each other, and they ultimately affect the cell death process [30]. In this study, to verify the role of ROS and ERS in the apoptosis of septic CD4^+^ T cells, NAC and 4-PBA were used to block ROS and ERS, respectively, and CD4^+^ T cell apoptosis decreased in both groups.

When sepsis happened, there are two pathways that lead to systemic inflammation and oxidative stress: endogenous DAMPs and exogenous PAMPs (pathogen-associated molecular patterns). DAMPs are proinflammatory substances released upon host cell damage. As effective activators of body immune reaction that initiate and maintain damaged inflammatory responses, DAMPs can cause systemic inflammation, organ dysfunction, and even death [31]. CIRP, a newly discovered DAMP, recognized among different kinds of cells (e.g., T cells, B cells, and macrophages), is an important upstream molecular of ROS [17]. By blocking CIRP, we observed that ROS production and ERS occurrence were reduced, CD4^+^ T cell apoptosis decreased, and the survival rates of the septic mice improved. From these results, we conclude that ROS and ERS are involved in septic CD4^+^ T cell apoptosis and that ROS can cause ERS. The occurrence of ERS further mediates the damaging effect of ROS on CD4^+^ T cells and causes CD4^+^ T cell apoptosis.

mTOR is a sensitive and conserved cellular energy sensor. It is activated by various hormones and growth factors and has a key function in regulating cell metabolism, protein synthesis, growth, and differentiation [32]. Studies have highlighted mTOR's role in the energy metabolism of cells [33, 34]. When sepsis occurs, the body is in an inflammatory state caused by severe hypoxia and stress. Oxygen utilization and energy metabolism in the body's cells are both affected, and ROS production increases. ROS accumulation affects the translation, synthesis, and processing of proteins, leading to the occurrence of ERS and, ultimately, cell apoptosis [30]. In the body's fierce anti-infection process, lymphocytes are undoubtedly the first line of defense to bear the brunt of the infection, as evidenced by the apoptosis of large numbers of lymphocytes and the subsequent immunosuppression. The flow cytometry results from the present study are consistent with this. Sepsis therefore causes lymphocyte apoptosis and immunosuppression, and ROS accumulation aggravates the lymphocyte energy metabolism disorder. As a crucial part of energy metabolism regulation, what role does mTOR play in this process? To answer this question, we explored the role of mTOR in the ERS-related CD4^+^ T cell apoptosis mediated by ROS. We explored the role of mTOR by constructing septic models with T cell-specific mTOR/TSCI-KO gene knockout mice. Our results showed that CD4^+^ T cell apoptosis in the Lck-mTOR+CLP group decreased, while CD4^+^ T cell numbers increased. Significantly longer survival time was observed in mice that were treated with Lck-mTOR+CLP. This indicates that mTOR is activated by ERS to affect CD4^+^ T cell apoptosis during sepsis.

Our results showed that when ERS occurred, mTOR is activated, as was revealed by two main results. (1) The inhibition of ERS by 4-PBA downregulated mTOR expression in parallel with decreased CD4^+^ T cell apoptosis. (2) The occurrence of ERS was unaffected when mTOR and TSC1 (the inhibitor in the mTOR signaling pathway) genes were knocked out. Consistently, the study by Kato et al. showed that ERS rapidly activated mTORC1 in a mouse model of renal tubular injury, and mTORC1 is rapidly activated with the treatment of ERS inducers thapsigargin and tunicamycin [10]. However, some researchers believe that activating mTOR induces ERS. For example, Ozcan et al. reported that the loss of TSC1 or TSC2 (the inhibitor of mTOR) and the subsequent activation of mTORC1 led to ERS, thereby making the cells more susceptible to apoptosis and death [35]. The reasons for the inconsistencies between the present study and others may be related to the different disease models and different stimulus methods used to induce ERS. For example, in their article, Ozcan et al. pointed out that ERS was provoked by drug stimulation on isolated cells and that this may differ from the real situation in vivo. In contrast, our mouse model of sepsis was used to induce ERS in a manner as close as possible to the pathophysiological process of severe infection. The ERS in our study was more serious ERS and led to cell apoptosis and ultimately death in the host. The flow cytometry and survival curves confirmed the lymphocyte apoptosis and the timing of host death. Relevant studies have also shown that ERS upregulates the expression of mTORC1 and that the cytotoxicity of ERS is significantly related to mTORC1 activation [10^,^^36^]. Therefore, our results suggest that mTOR is involved in regulating ERS-induced apoptosis, although the specific mechanisms involved require further exploration.

Herein, we found that mTOR is involved in ROS-related energy metabolism disorders. Through the intervention of mTOR, the stress and damaged state of CD4^+^ T cells in sepsis were improved, CD4^+^ T cell apoptosis was reduced, and the prognosis of the host was improved. These results provide a possible treatment avenue for improving the immune status of patients with sepsis. However, some aspects of our study require further investigation. First, when ERS occurs, there are three known signaling pathways that sense and relieve the occurrence of ERS; namely, IRE1, PERK, and ATF6 sensory pathways. In future research, we will focus on the precise sensing signal pathways to investigate how mTOR perceives and regulates ERS-related CD4^+^ T cell apoptosis. Second, further study is warranted to determine the exact mechanism of the mTOR pathway leading to CD4^+^ T cell apoptosis, including the detection of more specific downstream cytokine levels.

## 5. Conclusions

As far as we know, the upstream mechanisms by which mTOR regulates lymphocyte apoptosis in sepsis have not been elucidated. We explored the role of mTOR in CD4^+^ T cell apoptosis induced by ERS. We found that ROS accumulation in sepsis led to ERS occurrence and that the mTOR pathway operating downstream of ERS induced CD4^+^ T cell apoptosis. By inhibiting mTOR, CD4^+^ T cell apoptosis was reduced, and the prognosis of the septic mice was improved. This indicates that mTOR participates in and regulates ROS-mediated ERS-related CD4^+^ T cell apoptosis in sepsis, raising the possibility of mTOR becoming a new targeted treatment strategy for alleviating CD4^+^ T cell apoptosis and improving the immune status of those experiencing sepsis.

## Data Availability

Data are available on request.
