# Correction to “mTOR Modulates the Endoplasmic Reticulum Stress‐Induced CD4+ T Cell Apoptosis Mediated by ROS in Septic Immunosuppression”

**DOI:** 10.1155/mi/9767604

**Published:** 2026-02-09

H. Wang, J. Chen, G. Bai, W. Han, R. Guo, and N. Cui, “mTOR Modulates the Endoplasmic Reticulum Stress‐Induced CD4+ T Cell Apoptosis Mediated by ROS in Septic Immunosuppression,” *Mediators of Inflammation*, 2022, 6077570, https://doi.org/10.1155/2022/6077570.

In the article, there are errors in Figures 5 and 6 introduced during manuscript preparation. Specifically:

• The CLP and Lck‐‐TSC1‐CLP panels of Figure 5c are incorrect.
• The ß‐actin bands in Figure 6b,c are erroneously identical.

After assessment of the concerns and author’s raw data, the editorial board has agreed to a correction of Figures 5 and 6. Please find the correct Figures 5 and 6 below:

Figure 5Role played by mTOR in ERS and CD4+ T cell apoptosis. CD4+ T cell apoptosis percentage was assessed by flow cytometry (a). Left panel: gating strategy for apoptosis cells; right panel: percentage of apoptotic cells in the left panel. Graphs show means ± SD, four mice per group. mRNA expression level of BIM in CD4+ T cells was analyzed by RT‐PCR (b). Representative images of ER in CD4+ T cells from CLP, LCK‐TSC1 + CLP, and Lck‐mTOR + CLP mice, as observed by electron microscopy (c). Data are mean ± SD, *n* = 4, analyzed using one‐way ANOVA. Blue arrows represent the normal‐sized ER. Red arrows represent dilation of the ER. Scale bars = 0.5 μm.

**Figure:**
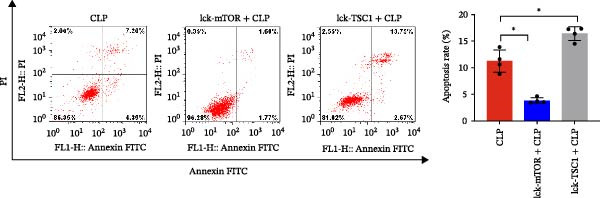
(a)

**Figure:**
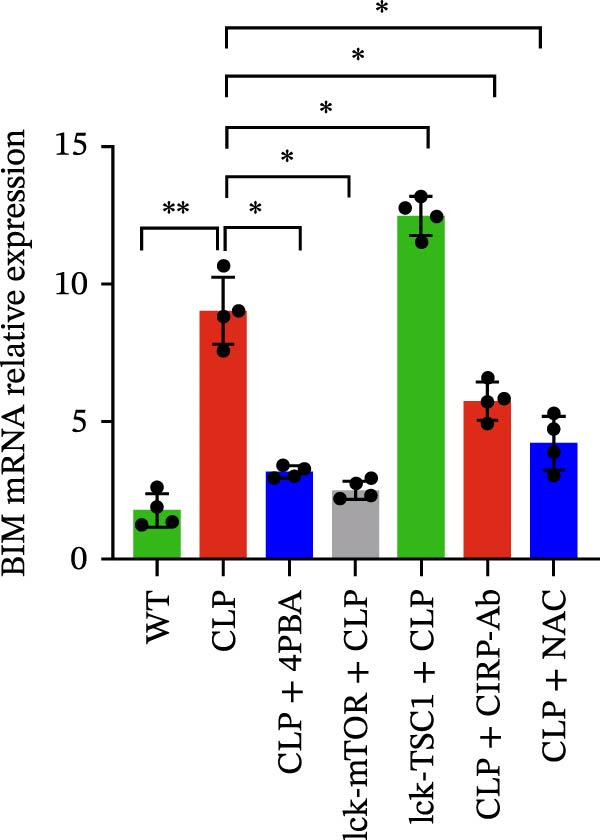
(b)

**Figure:**
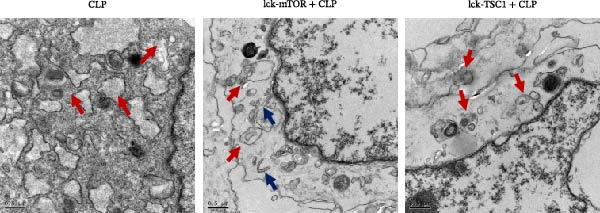
(c)

Figure 6CD4+ T cells were assessed for the expression of mTOR pathway‐associated proteins, ERS‐associated proteins, and apoptosis‐associated proteins in WT, LCK‐mTOR, LCK‐TSC1, CLP, CLP + CIRP‐Ab, CLP + NAC, LCK‐TSC1 + CLP, and Lck‐mTOR + CLP mouse groups. Data are mean ± SD. Number of mice per group = 4 (one‐way ANOVA Tukey’s post hoc test). CD4+ T cells were purified from CIRP‐Ab‐treated mice, NAC‐treated mice, TSC1 knockout mice, and mTOR mouse spleen lymphocytes. Total proteins were western blotted to identify the expression patterns of mTOR pathway proteins (P‐mTOR, mTOR, p70s6k, p‐p70s6k, P‐4EBP, and 4EBP) (a); CIRP and ERS‐associated proteins (GRP78 and CHOP) (b); and apoptosis‐related proteins (caspase‐3, Bax, and Bcl‐2) (c).

**Figure:**
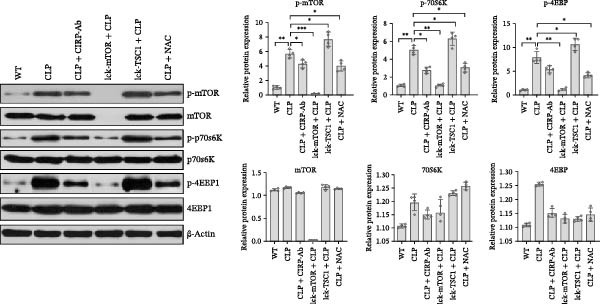
(a)

**Figure:**
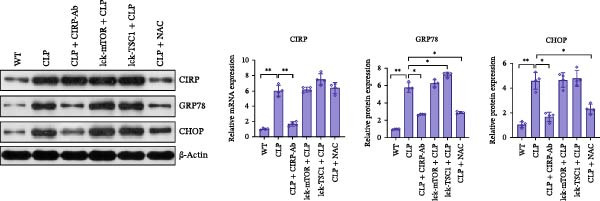
(b)

**Figure:**
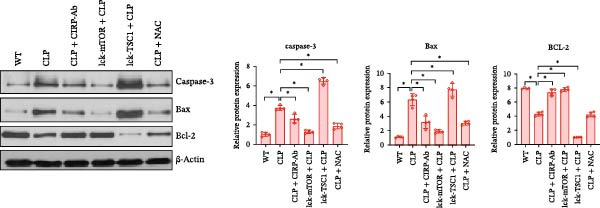
(c)

We apologize for these errors.

